# The role of glial cells in neuroinflammation in the context of Alzheimer’s disease

**DOI:** 10.1002/alz.085661

**Published:** 2025-01-03

**Authors:** Rafaella de Lima Corrêa Ferreira, Luana Heimfarth, Sergio T Ferreira, Felipe C Ribeiro

**Affiliations:** ^1^ Federal University of Rio de Janeiro, Rio de Janeiro, Rio de Janeiro Brazil; ^2^ Federal University of Rio de Janeiro, Rio de Janeiro, RJ Brazil

## Abstract

**Background:**

Alzheimer’s disease (AD) stands as the predominant form of dementia worldwide. The pathogenesis of AD encompasses elevated brain levels of amyloid‐β oligomers (AβOs), recognized as central neurotoxins linked to AD. The accumulation of AβOs is neurotoxic, resulting in detrimental effects such as synapse loss, mitochondrial dysfunction, and impairment of proteostasis mechanisms. Neuroinflammation, particularly the activation of microglia and astrocytes to pro‐inflammatory states, has been implicated in the pathogenesis of various neurodegenerative diseases. This study aimed to investigate the role of microglia and astrocytes within the framework of AD pathophysiology.

**Method:**

Here, we explored changes in morphology and function of glial cells in experimental models of AD, assessing (1) *in vitro* activation of microglia and astrocytes induced by AβOs, and (2) *in vivo* microglia and/or astrocytic activation in mice receiving an intracerebroventricular (icv) infusion of AβOs. Analysis of cytokine expression and glial markers was conducted using qPCR, ELISA, and fluorescence microscopy.

**Result:**

Initial results suggest an upregulation of glial activation markers, such as GFAP and F4/80, and complement immune system markers such as C1q, seven days post icv infusion of AβOs. No significant differences were observed in TNF‐α expression. Immunofluorescence analyses further indicate heightened nuclear translocation of NFkB in microglia cultures exposed to AβOs for 3 hours.

**Conclusion:**

The results align with a pivotal role of glial cells in AD pathogenesis, showcasing activated glial activity in both *in vitro* and *in vivo* models. In the context of chronic processes, these cells may persist in an activated state, releasing cytokines, complement components, and chemokines, potentially contributing to Aβ production and accumulation, thereby exacerbating pathology.

